# From personality types to social labels: the impact of using MBTI on social anxiety among Chinese youth

**DOI:** 10.3389/fpsyg.2024.1419492

**Published:** 2024-09-04

**Authors:** Wenjing Wu, Wenzhu Hao, Gao Zeng, Weiyi Du

**Affiliations:** School of Journalism and Communication, Shanghai International Studies University, Shanghai, China

**Keywords:** MBTI, social labels, personality types, social anxiety, Chinese youth

## Abstract

**Introduction:**

As the Myers-Briggs Type Indicator (MBTI) gains popularity among Chinese young people, it has undergone a gradual transition from being perceived as a personality assessment tool to being regarded as a social label. The objective of this study was to ascertain whether the use of the MBTI as a social label has an impact on social anxiety among Chinese youth groups.

**Methods:**

A questionnaire survey was conducted on social media platforms to recruit Chinese youth aged 18 to 35. A total of 247 males and 222 females participated in the study, and the data was analyzed quantitatively using SPSS software and the Process macro plugin.

**Results:**

The study found no strong correlation between MBTI as a social label and social anxiety. Moreover, this study introduced ego identity, belonging, and impression management as mediating variables and found that, under the influence of ego identity and impression management, the use of MBTI has a significant impact on social anxiety.

**Discussion:**

The research reveals the complex role of MBTI among Chinese youth and provides a new perspective for understanding the impact of online social labels on the mental health of youth groups. Of course, this study also has limitations in terms of sample size and variable control. Future research should expand the sample size, introduce more potential influencing factors, and further validate and expand the existing conclusions.

## 1 Introduction

The Myers-Briggs Type Indicator (MBTI) is a measurement tool for psychological type theory proposed and developed by Isabel Briggs Myers and her mother Katharine Cook Briggs. It has gained widespread international influence and application prospects, particularly in areas such as leadership potential (Pestana and Codina, 2019; Pestana and Codina, 2020), management behavior (Furnham and Stringfield, 1993; Wu et al., 2011), and career planning (McCaulley and Martin, 1995). The design of MBTI is primarily based on the psychological type theory proposed by Jung (1971), who believed that people’s psychological tendencies can be divided into two basic attitudes: extraverted [E] and introverted [I], which further extend to four psychological functions, namely thinking [T], feeling [F], sensing [S], and intuition [N]. Twenty years after Jung proposed the psychological type theory, Myers and Briggs introduced the fourth dimension: judging [J] and perceiving [P] (Quenk, 2009). The MBTI test separates human personality types into four dimensions and ultimately combines them into 16 personality types.

Although the MBTI still has limitations in psychological measurement aspects such as reliability and usefulness (Boyle, 1995), it remains popular among Chinese youth. As of December 16, 2023, the real-time popularity of MBTI-related topics on the Xiaohongshu app (China’s version of Instagram) in the past 180 days has reached 31.2817 million. Discussions on this topic mainly revolve around interpersonal communication, indicating that MBTI has transformed from a personality type test to a means of interpersonal interaction. Recent studies have also found that some researchers have begun to focus on the importance of MBTI personality types in online social activities (Zhang, 2024) and how the spread of MBTI has become a social culture (Wang et al., 2024). However, their research lacks a quantitative perspective to explain this issue. With the popularity of online social media, people can communicate with others through social software (such as Twitter, Facebook, Instagram) anytime and anywhere without leaving their homes. However, as the frequency of social media use increases, people are gradually experiencing more intense social anxiety (Yang et al., 2023).

Therefore, this study, using MBTI as an entry point, will explore its impact on social anxiety among Chinese youth through a survey method. Additionally, the use of MBTI may promote ego identity and further trigger group identity, which will also affect impression management made by individuals to conform to MBTI traits (Zhang, 2024). Therefore, this study will also explore “ego identity,” “belonging” and “impression management” as mediating variables.

## 2 Literature review

### 2.1 Using MBTI as a social label and social anxiety

Social Anxiety (SA) differs from general anxiety symptoms; it primarily occurs during interpersonal interactions and is characterized by intense fear of others’ evaluations in social situations (Morrison and Heimberg, 2013; Counsell et al., 2017). When social anxiety escalates to a certain degree, it can lead to Social Anxiety Disorder (SAD). People with SAD often feel shy when encountering strangers and may want to withdraw from unfamiliar social environments. They are often ambivalent in nature, desiring social interaction while avoiding it due to fears of being disliked, perceived as stupid, or boring (Stein and Stein, 2008). With China’s economic, cultural, and social development, social anxiety among Chinese youth is also increasing (Xin et al., 2022). Some scholars argue that urbanization and employment pressures have, to some extent, reduced interpersonal connections, disrupting China’s traditional acquaintance society and thereby increasing the likelihood of social anxiety (Chi and Xin, 2020; Xin et al., 2021).

The internet has become a crucial platform for interpersonal communication, and posting daily updates online has become a common habit among contemporary Chinese youth. Among these practices, the use of MBTI is gradually replacing the previous 12 astrological types, becoming an important social label for Chinese youth on the internet. There is a difference between social tags and social labels on the internet. Social tags mainly emphasize the technical aspects of social media, fulfilling various user needs such as information classification, search and navigation, and information extraction (Suchanek et al., 2008). However, as social media evolves, more people are starting to label themselves and others, referred to as social labels. Social label theory focuses on the role of these labels in criminal and deviant behavior; once labeled, individuals are prone to eliciting negative stereotypes from themselves and others (Becker, 1963). Social label theory emphasizes how societal environments, by defining or stereotyping individuals as deviants, can trigger a series of deviant behaviors (Bernburg, 2009). In other words, labels are essentially constructs of social norms; they do not inherently exist (Becker, 1963). In social label theory, once labeled as a norm-breaker, individuals are tagged as “deviants.” In practice, MBTI personality types also serve as a form of social label; when individuals exhibit personality traits differing from their MBTI type, they may be labeled as “deviants” by the MBTI-using community, leading to gradual isolation. This intensification may increase social anxiety as they are tagged with undesirable labels (Stein and Stein, 2008). Therefore, to embody their MBTI personality type, individuals must conform to behaviors aligned with this type, driving themselves to integrate into the group and gain acceptance. It can be argued that to cater to group needs, individuals may experience social anxiety. However, this theoretical derivation remains untested. To fill this gap, the study attempts to validate this derivation. Thus, the first hypothesis of the study is:

*H1*: Using MBTI as a social label positively influences social anxiety.

### 2.2 Using MBTI as a social label, ego identity, and social anxiety

The concept of ego identity was proposed by Erikson (1956), who discussed the formation of self-identity within the framework of personality development. Although Erikson did not provide a clear definition of ego identity, it is generally understood that it needs to be discussed within a psychoanalytic perspective (Bourne, 1978). The formation of ego identity is a crucial aspect of personality development. To further measure it, Marcia (1966) proposed the dimensions of exploration and commitment, categorizing ego identity into four types based on whether exploration leads to commitment: achievement, moratorium, foreclosure, and diffusion. Marcia’s theory provided the possibility for empirical research on Erikson’s ego identity theory, leading to the development of many semi-structured interview techniques and standardized measurement techniques, and transitioning Erikson’s identity theory into a clearly verifiable theory. Previous research has demonstrated a positive correlation between using MBTI and ego identity (Hua and Zhou, 2023), suggesting that external personality information can influence ego identity to some extent. When MBTI serves as a social label, it provides a clear symbol for achieving ego identity. These symbols from the external world enable individuals to better achieve ego identity (Brown, 1998).

In the context of the internet, there is research on the relationship between ego identity and social anxiety. The findings reveal that for male youth, there is a positive correlation between immature identity status, frequent internet use, and social anxiety, while no such relationship is found for female youth (Mazalin and Moore, 2004). Once a mature identity status is achieved through MBTI among youth, is there still a positive correlation with social anxiety? Perhaps achieving ego identity through MBTI may reduce social anxiety, but if confined within the MBTI social label, will ego identity still reduce social anxiety? Based on theoretical derivations and the emergence of new questions, the study suggests the following hypotheses:

*H2: Using MBTI as a social label positively influences ego identity.*

*H3: Ego identity achieved through MBTI positively influences social anxiety.*

*H4: Ego identity plays a mediating role between using MBTI as a social label and social anxiety.*

### 2.3 Using MBTI as a social label, belonging, and social anxiety

Belonging is considered a subjective sense of value and respect, established on the basis of shared experiences, beliefs, or personal characteristics (Mahar et al., 2013). Belonging is primarily understood from the perspective of interpersonal relationships as an important measure of whether there is a lack of mental health (Anant, 1966). When belonging is lacking, it is likely to trigger anxiety in individuals (Baumeister and Tice, 1990). Belonging, as a universal human need, serves to establish bonds with others. Achieving belonging involves two criteria: the need for frequent and enjoyable interactions with a few others and the requirement for these interactions to occur within a temporarily stable, enduring, and mutually beneficial emotional framework (Baumeister and Leary, 1995). Using MBTI as a social label has the potential to enhance belonging, primarily due to MBTI’s classification function, which divides people into 16 personality types. Classification helps individuals understand their social environment and plays a positive role in guiding individual behavior within it (Bruner, 1957; Bodenhausen et al., 2003). Classification also facilitates understanding the relationships between one’s own group and other groups and promotes the search for shared values among groups (Galinsky et al., 2003). Through MBTI personality types, people can more easily distinguish themselves from others and ultimately achieve group categorization based on personality types. Because this categorization is based on personality types, there is similarity or proximity among group members, which helps people establish a bond and form relatively stable and enjoyable interactions (Baumeister and Leary, 1995). Furthermore, classification defines a common social identity for individuals, which helps foster a sense of belonging and unity, promoting trust and cooperation among people (Claridge, 2020). Groups formed by shared identities prevent individuals from being isolated, but this also means that individuals must commit to group expectations and obligations, strive to serve group interests, and avoid actions that undermine group goals (Ellemers et al., 2002; Claridge, 2020). Therefore, individuals who use MBTI as a social label are compelled to behave in accordance with group expectations and obligations to avoid being detached from the group, which indirectly leads to social anxiety. It can be argued that while belonging may alleviate social anxiety to some extent, when assigned a social label, this belonging may lead to deeper social anxiety. To further validate the above theoretical derivation, the study proposes the following hypotheses:

*H5: Using MBTI as a social label positively affects belonging.*

*H6: The belonging obtained from using MBTI positively affects social anxiety.*

*H7: This belonging plays a mediating role between using MBTI as a social label and social anxiety.*

### 2.4 Using MBTI as a social label, impression management, and social anxiety

Humans have a universal and ongoing concern about self-presentation, sometimes acting to leave a particular impression on others (Leary, 1995). In interpersonal relationships, impression management controls the formation of others’ impressions by creating an image that others expect to see Leary and Kowalski (1990). Through interpersonal interactions, impression management can establish, maintain, or refine an individual’s image in the minds of others (Tedeschi, 2013). Impression management consists of two processes: the motivation for impression management and impression construction. The former emphasizes the extent to which people want to control others’ perceptions of them, while the latter refers to the type of impression people try to construct (Leary and Kowalski, 1990). There are mainly five strategies of impression management: ingratiation, self-promotion, exemplification, supplication and intimidation, which may not necessarily yield positive effects (Jones and Pittman, 1982; Turnley and Bolino, 2001). When MBTI is used as a social label, it is likely to generate motivation for impression management during interpersonal interactions (Zhang, 2024). The motivation for impression management arises from the goal relevance of the impression, the value of the desired goal, and the discrepancy between the desired image and the current image (Leary and Kowalski, 1990). Past understandings of impression management were often based on the need to deceive others or meet social expectations, but research has confirmed that impression management in social interactions is not for deception but rather self-control oriented towards interpersonal relationships (Uziel, 2010, 2014). Previous studies have shown that individuals with high levels of social anxiety tend to have stronger self-control abilities during interactions with others (Kashdan et al., 2011). When self-control weakens during or after social interactions, it is positively correlated with social anxiety (Kashdan et al., 2011; BlaCkhart et al., 2015). If individual self-control is for the sake of impression management, it will bring additional burdens to social anxiety (Finkel et al., 2006; BlaCkhart et al., 2015). However, when focusing on MBTI as a social label, is it also the case that self-control in impression management, within the context of interpersonal relationships, mediates social anxiety? This question remains unclear. Therefore, the study proposes the following hypotheses:

*H8: Using MBTI as a social label positively affects self-control in impression management.*

*H9: The self-control in impression management obtained from using MBTI positively affects social anxiety.*

*H10: This self-control in impression management plays a mediating role between using MBTI as a social label and social anxiety.*

**Figure 1:**
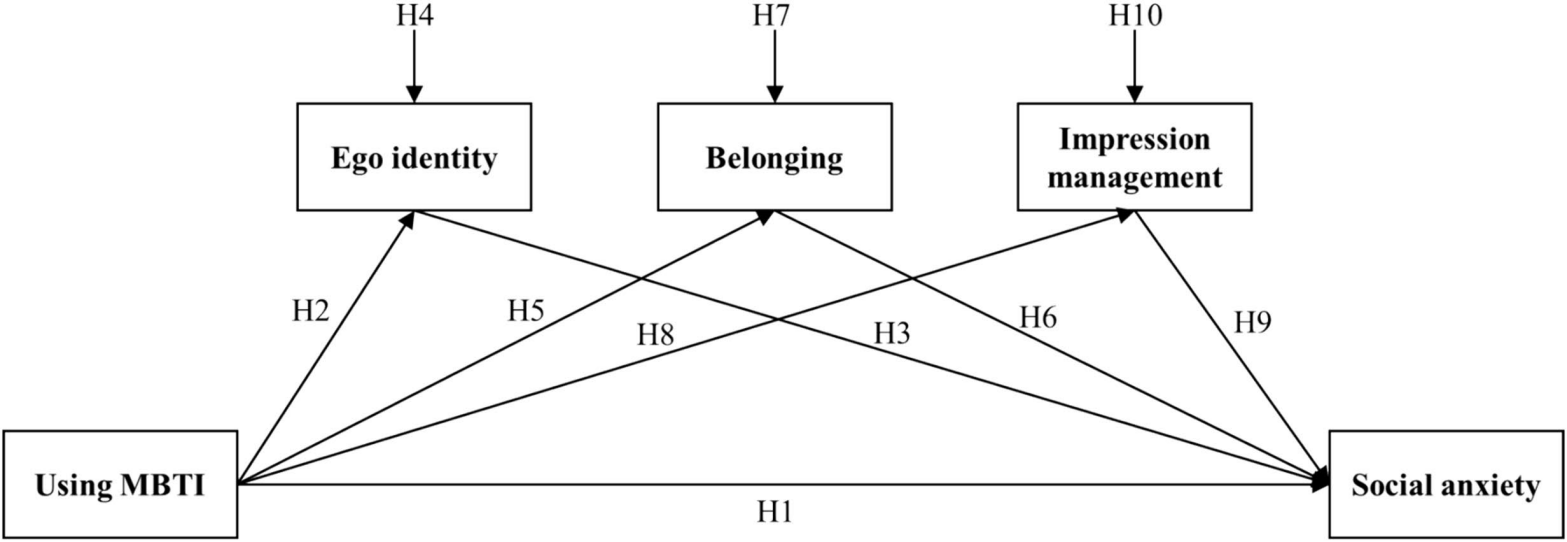
Hypotheses proposed in the study.

See Figure 1 for the research model diagram.

## 3 Research methods and data collection

### 3.1 Research design

In summary, ego identity, belonging, and impression management are the research dimensions that may lead to social anxiety among youth groups during the use of MBTI. This study adopts the questionnaire survey method, which utilizes questionnaires as a tool to collect data and is widely used in current social surveys (Moser and Kalton, 1971). The study employs ego identity, belonging, and impression management as mediating variables to explore the direct and indirect relationships between MBTI use and social anxiety among youth groups through questionnaire surveys. The survey targets young social media users aged 18 to 35. Since there is no unified standard for defining the age range of youth groups, and according to the World Health Organization’s definition of youth aged 18–44, this study selects participants aged 18–35 based on China’s national conditions and previous research on youth groups (Liu et al., 2022).

Participants in the study were mainly recruited through the dissemination of information on social media platforms widely used in China, such as WeChat, Weibo, QQ, Xiaohongshu, Douban, etc. After eliminating invalid questionnaires with obvious indications of minimal MBTI use, short response times, and low answer discrimination, a total of 469 valid questionnaires were obtained, with an effective rate of 84.01%. The sample mainly covers relatively economically developed regions in China, such as Shanghai, Guangdong, and Zhejiang, and includes 247 males (52.7%) and 222 females (47.3%). Their age groups are 18–25 years old (29.2%), 26–30 years old (43.7%), and 31–35 years old (27.1%). The study also investigated their educational background. Specific demographic variables are shown in Table 1.

**Table 1:** Basic information of participants.

Basic information	Category	Number	Percentage (%)	Cumulative percentage (%)
Gender	Male	247	52.7	52.7
	Female	222	47.3	100.0
Age group	18–25 years old	137	29.2	29.2
	26–30 years old	205	43.7	72.9
	31–35 years old	127	27.1	100.0
Education level	Junior high school or below	19	4.1	4.1
	High school/vocational school	54	11.5	15.6
	Junior College	147	31.3	46.9
	Undergraduate	165	35.2	82.1
	Postgraduate or above	84	17.9	100.0

### 3.2 Measurement scales

#### 3.2.1 MBTI usage measurement

We adopted the Contagion-Behavioral Response (CBR) scale to measure the usage of MBTI among Chinese youth groups. The CBR scale measures reporting behaviors related to reading and basic application of MBTI information (Lopez et al., 2021), without covering a deeper understanding of MBTI information. Therefore, this study modified some items to measure individuals’ understanding of MBTI and the frequency of usage behaviors such as liking, following, and sharing, to better understand participants’ MBTI usage. Participants were required to indicate their level of agreement with each item on a five-point Likert Scale (1 = Never, 5 = Always), and the degree of MBTI usage was assessed by averaging all scores.

#### 3.2.2 Ego identity measurement

Adolescence is a critical juncture for establishing cognition of ego identity and social identity. Currently, MBTI has become a widely influential personality analysis model. Although its reliability and validity are questioned in academic circles, as a new form of social label, it has a significant impact on adolescents’ social ego identity, further affecting social anxiety among youth. Serafini and Adams (2002) constructed an identity scale based on Loevinger (1957) test method to measure the five identity functions proposed by Adams and Marshall (1996), and verified the validity of the scale from internal consistency, structural validity, and external functionality. We adopted this scale in this study to explore whether participants’ self-cognition changed after using MBTI (1 = Never, 5 = Always).

#### 3.2.3 Belonging measurement

This study references the social connection scale and social assurance scale developed by Lee and Robbins (1995) based on self-psychology theory. These two scales can measure the social belonging of youth groups in MBTI usage from different dimensions such as psychological distance and group consciousness (1 = Strongly agree, 6 = Strongly disagree). The reliability estimates of the scales are 0.91 and 0.82, respectively, indicating good reliability.

#### 3.2.4 Impression management measurement

In the process of labeling MBTI among youth groups, basic social needs often stimulate their motivation for impression management. The two-component model of impression management suggests that impression management is a two-stage process, including impression management motivation and impression construction. Impression management motivation is defined as “the extent to which people are motivated to control how others perceive them” (Leary and Kowalski, 1990). In the process of using MBTI labels for social interaction, youth groups are likely to experience social anxiety based on the need for impression management. This study drew on the self-control measurement scale developed by Tangney et al. (2004), which improves upon the self-control questionnaire proposed by Brandon et al. (1990) by incorporating individual difference factors into the measurement of self-control, demonstrating good reliability. Participants in this study were required to make internal judgments and choices related to impression management dimensions based on their true thoughts after using MBTI (1 = Strongly disagree, 5 = Strongly agree).

#### 3.2.5 Social anxiety scale

The use of MBTI among youth groups is influenced by social anxiety to some extent. Different personality classifications can easily lead to phenomena such as social group division and individual isolation. Schlenker believes that people’s concern about receiving unfavorable evaluations from others is related to many social psychological phenomena, including conformity, prosocial behavior, self-presentation, self-serving attribution, social anxiety, self-handicapping, attitude change, etc. (Schlenker and Leary, 1982). The Fear of Negative Evaluation (FNE) scale proposed by Watson and Friend (1969) is the most commonly used measure to determine the degree of fear of receiving negative evaluations from others, with social anxiety being an important indicator. However, the utility of this scale is limited by its length. Therefore, Mark R proposed a short version of the scale (Leary, 1983), which has only 12 items but is highly correlated with the original scale. This study borrows from this scale and modifies the items according to the participants’ context, measuring the degree of social anxiety in MBTI usage from the perspective of the influence of others’ evaluations on self-evaluation (1 = Strongly disagree, 5 = Strongly agree).

### 3.3 Data analysis

The data organization tool for this study was Excel, while the data analysis tools were SPSS 27.0 and the Process 4.1 macro plugin developed by Hayes (2013). Due to inconsistencies in the scales of individual variables, the data were first standardized using z-score normalization in SPSS 27.0 before analyzing. Subsequently, all items underwent reliability and validity tests. Based on the standardized item data, the average value of different variables were calculated, followed by the computation of correlations between variables. Finally, model 4 in the Process 4.1 macro plugin was used for parallel mediator variable analysis. For all mediator variables, the Bootstrap test was employed to further verify their mediating effects.

#### 3.3.1 Reliability and validity tests

The items selected for the study were validated in the researcher’s papers and exhibited good reliability and validity (Leary, 1983; Lee and Robbins, 1995; Serafini and Adams, 2002; Tangney et al., 2004; Lopez et al., 2021). To further examine the reliability and validity of the survey data, Cronbach’s Alpha coefficient, KMO value, and Bartlett’s Test of Sphericity were calculated using SPSS 27.0. The results showed that the Cronbach’s Alpha coefficients for all dimensions ranged from 0.867 to 0.955, all higher than the threshold of 0.7, indicating high internal consistency and ideal reliability of the scales in the questionnaire. The KMO values for all dimensions ranged from 0.888 to 0.977, all exceeding the threshold of 0.7, and the sig. Values for Bartlett’s Test of Sphericity were all 0.000, indicating high data validity. Meanwhile, due to the diverse sources of data in this study, a Harman’s single-factor analysis of variance was also conducted on the data. The results showed that the eigenvalues of 12 factors were greater than 1, and the variance explained by the first common factor was 26.23%, less than the standard value of 40%. Therefore, there was no common method bias, and the results were valid for subsequent data analysis. The results of reliability and validity analysis for each variable are shown in Table 2.

**Table 2:** Reliability and validity of each dimension of the scale.

Variable	Cronbach’s alpha	KMO	Bartlett’s test of sphericity
Using MBTI (UM)	0.867	0.888	sig. = 0.000
Ego identity (EI)	0.907	0.922	sig. = 0.000
Belonging (B)	0.892	0.935	sig. = 0.000
Impression management (IM)	0.913	0.977	sig. = 0.000
Social anxiety (SA)	0.955	0.974	sig. = 0.000

#### 3.3.2 Correlation analysis

To further investigate whether there were correlations between the dimensions, Pearson correlation coefficient measurements were conducted on all dimensions. As shown in Table 3, there were significant and positive correlations between Using MBTI, Ego Identity, Belonging, Impression Management, and Social Anxiety. Among them, Ego Identity had the highest correlation with Using MBTI (*r* = 0.754), followed by Belonging (*r* = 0.533), Social Anxiety (*r* = 0.324), and Impression Management (*r* = 0.295). It is evident that all variables were positively correlated, and both Ego Identity and Belonging had *r* values >0.5, indicating significant correlations with Using MBTI.

**Table 3:** Correlation analysis between variables.

Variables	1	2	3	4	5
1. Using MBTI (UM)	1				
2. Ego identity (EI)	0.754**	1			
3. Belonging (B)	0.533**	0.580**	1		
4. Impression management (IM)	0.295**	0.367**	0.337**	1	
5. Social anxiety (SA)	0.324**	0.402**	0.298**	0.593**	1

**p < 0.01.

#### 3.3.3 Regression analysis of the mediation model

The study used the Process macro program 4.1 developed by Hayes to perform regression analysis on the variables, selecting model 4. The regression analysis is shown in Table 4 (Hayes, 2013). The results indicated that Using MBTI had a significant positive effect on Ego Identity (*β* = 0.705, *p* < 0.001), Belonging (*β* = 0.530, *p* < 0.001), and Impression Management (*β* = 0.332, *p* < 0.001). Ego Identity (*β* = 0.272, *p* < 0.01) and Impression Management (*β* = 0.600, *p* < 0.001) had a significant positive impact on Social Anxiety. This suggests that Ego Identity and Impression Management partially mediate the relationship between Using MBTI and Social Anxiety. Therefore, hypotheses H2, H3, H5, H8, and H9 were supported, while H4 and H10 received initial support and required further testing. Hypotheses H1, H6, and H7 were not supported.

**Table 4:** Regression analysis of variable relationships.

Regression equation		Overall fit index		Significance of regression coefficients	
Dependent variable	Independent variable	*R* ^2^	*F*	*β*	*t*
EI	UM	0.568	614.571	0.705	24.790***
B	UM	0.283	184.831	0.530	13.5953***
IM	UM	0.087	44.583	0.332	6.677***
SA	UM	0.390	74.415	0.035	0.474
	EI			0.272	3.283**
	B			−0.002	−0.048
	IM			0.600	13.029***

**p < 0.01, ***p< 0.001.

**Figure 2:**
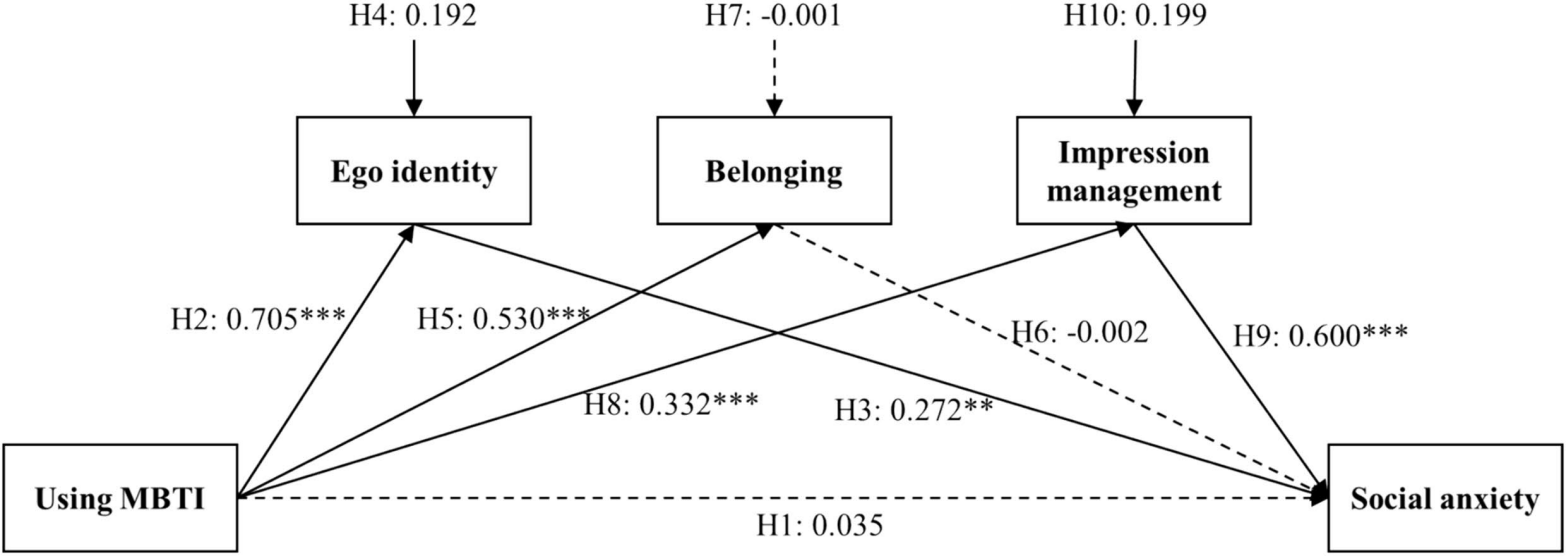
Model diagram of the association between using MBTI and social anxiety. Solid lines indicate supported hypotheses, and dashed lines indicate unsupported hypotheses. **p < 0.01, ***p < 0.001.

To further estimate the performance indicators of the model, a Bootstrap test was conducted on the mediator variables, as shown in Table 5. In the process where Using MBTI affects Social Anxiety through Ego Identity, Belonging, and Impression Management, both Ego Identity and Impression Management did not include 0 in the Bootstrap 95% confidence interval, indicating significant mediating effects of these two variables. Belonging included 0, further confirming that hypothesis H7 was not supported. Therefore, hypotheses H4 and H10 were supported. According to the research data, the mediation model effect is shown in Figure 2.

**Table 5:** Analysis of mediation effects.

	Indirect effect value	Bootstrap SE	Bootstrapping 95% CI		Relative indirect effects (%)
			Lower-bound	Upper-bound	
Total mediating effect	0.390	0.072	0.249	0.530	91.80%
EI	0.192	0.065	0.065	0.316	45.23%
B	−0.001	0.032	−0.064	0.063	−0.35%
IM	0.199	0.029	0.142	0.258	46.92%

## 4 Discussion and conclusion

### 4.1 Analysis of research findings

#### 4.1.1 The relationship between using MBTI and social anxiety

The correlation between using MBTI as their social label and social anxiety in Chinese youth is weak, and the results from the Process 4.1 macro plugin are also insignificant. This outcome roughly confirms that the relationship between the two is not close, and it is clear that using MBTI as a social label does not lead to social anxiety. In previous research, scholars measured whether using MBTI by adolescent groups would bring mental health issues, and their results showed that it could enhance the level of subjective well-being, thereby reducing anxiety and depression (Hua and Zhou, 2023), but they did not discuss it in social interactions. The results of this study further indicate that using MBTI as a social label may not bring any harm. The possible reason for this outcome is that during the long-term use by Chinese youth, MBTI has primarily served to further strengthen self-cognition, without excessive focus on whether interactions with others would bring adverse consequences. This is also an important reason why social anxiety is difficult to arise. In other words, simply acquiring and using a social label may not impact social anxiety.

#### 4.1.2 Differences in the mediating effects of ego identity, belonging, and impression management

Due to the insignificance of the direct effect, the mediating effects influencing social anxiety become crucial. Next, we discuss several mediating effects proposed in the research hypothesis.

##### 4.1.2.1 Significant mediating effect of ego identity

Previous research clearly indicates that using MBTI will have a positive impact on ego identity, and immature self-cognition is more likely to trigger social anxiety (Mazalin and Moore, 2004; Ruth, 2013; Hua and Zhou, 2023). Does this mean that acquiring ego identity through MBTI will alleviate social anxiety to some extent? The survey data suggests that this may not be the case. The emergence of MBTI has gradually clarified ego identity. The past self may have been relatively chaotic, with greater interpretive space, where different viewpoints would be seen as individual differences rather than linked to personality types. However, after ego identity is clarified, it may not immediately bring excessive harm, but long-term interactions could potentially disrupt this relatively stable state. Chinese youth primarily use MBTI as a social label on social media, engaging in frequent and prolonged interactions with others. It is highly likely that some personality traits proposed by MBTI cannot fully explain individual behavior, even leading to doubts about MBTI. Ultimately, MBTI still has limitations in reliability and usefulness (Boyle, 1995). Individuals in this state may experience a relatively contradictory state: Can MBTI as a social label truly help me understand myself and others? Is my interaction with others based on MBTI still meaningful? Or, having roughly figured out this person through MBTI, will they not have a good outcome with me? Is it necessary to socialize with them? Is judging a person through MBTI too one-sided? The emergence of these questions will influence the generation of social anxiety to some extent.

##### 4.1.2.2 Significant mediating effect of impression management

From the perspective of interpersonal relationships, impression management is more about self-control. When self-control weakens, it is unsurprising that social anxiety arises (Uziel, 2014). The data clearly shows that impression management has a relatively large impact on social anxiety, and its mediating effect is the strongest. This is primarily because, while MBTI serves as a social label, it also becomes a shackle in interpersonal interactions. People need to self-control according to the impressions that most people have of this personality label. Even if these impressions are stereotypes, prejudices, and discriminations, they have to do so (Gilbert et al., 1998; Fiske et al., 2009). This is because those who hold these impressions do not think there is anything wrong with them, as they avoid cognitive dissonance by constantly denying new cognition (Aronson, 1969; Jonas et al., 2001). However, from a personal perspective, these impressions will exert greater pressure on individuals. They may not be like what is described in MBTI but are forced to become someone with that personality trait. Individuals with stronger self-cognition may think, “This is not the real me, but I have to abide by these so-called ‘rules’ when socializing with others.” In this case, the increasing social pressure will further lead to social anxiety. That is, in social interactions with others, the mask needs to be present all the time. Once self-control weakens and the mask breaks, it will lead to others’ dislike. It is out of this concern that people have to pretend and control themselves, which also reflects the impact of impression management on social anxiety.

##### 4.1.2.3 Insignificant mediating effect of belonging

In traditional Chinese beliefs, social interaction is a process of gaining energy. It helps individuals seek solace from groups and feel happiness and joy. That is, people desire to socialize with others and acquire a sense of belonging (Baumeister and Leary, 1995). By using MBTI as a social label, people can quickly find their unique groups, thereby alleviating the possibility of social anxiety to some extent. The research hypothesis, when proposing this question, focused on people’s desire to maintain social interactions and the subsequent induction of social anxiety under a strong sense of belonging. However, the research hypothesis may have neglected the factors of accelerated urbanization and increased personnel mobility in China. A large number of Chinese youth, due to work, study, life, and other needs, have to leave familiar societies and enter unfamiliar ones. This has also led to relatively weak connections between people. Using MBTI can establish a sense of belonging, thereby escaping the state of being a relatively isolated individual. This also makes people more urgent to find suitable organizations or groups than to worry about social anxiety. Organizations formed based on personality labels also achieve a sharing of responsibilities to some extent. When individuals feel disliked, even foolish or boring, people are likely to attribute it to this personality label group rather than criticize the individual. In other words, by finding a group that provides a sense of belonging, individual responsibilities are shared by group members, leading to the “Diffusion of Responsibility” phenomenon (Darley and Latané, 1968). The above analysis clarifies the important reason for the insignificant mediating effect of belonging.

### 4.2 Research value

#### 4.2.1 Theoretical value

This research considers MBTI as a social label and explores its impact on social anxiety and the underlying mechanisms. Past research on MBTI at the social level mostly remained at theoretical speculation, often lacking empirical data support (Wang et al., 2024; Zhang, 2024). This study further explores the association mechanisms between using MBTI in social interactions and ego identity, belonging, impression management, and social anxiety from an empirical perspective. However, does frequent use of MBTI as a social label bring harm? To further investigate this issue, this research explores social anxiety as the dependent variable. This is primarily due to the increasing prevalence of social anxiety among Chinese youth (Xin et al., 2022). Will using MBTI become a new factor contributing to social anxiety among Chinese youth? Although the results do not support the hypothesis that directly using MBTI as a social label impacts social anxiety, they confirm that the mediating effects of ego identity and impression management will influence social anxiety. This research also contributes to viewing MBTI from different perspectives and provides a theoretical foundation for research on the social mental health of youth groups. Furthermore, the proposition of the impact of MBTI-based social interactions on social anxiety further confirms the strong external validity of different variables and dimensions.

#### 4.2.2 Practical value

Since MBTI as a social label may exacerbate or alleviate social anxiety in specific contexts, this helps mental health professionals and social media platforms formulate more precise intervention measures. Mental health professionals can provide more personalized psychological guidance to youth groups, thereby guiding them to correctly understand and use MBTI and reduce unnecessary psychological pressure. Social media platforms can optimize content recommendation and user interaction mechanisms to reduce social anxiety caused by the abuse of social labels.

### 4.3 Limitations and research prospects

Although this research has achieved certain outcomes, there are also some limitations. First, the diversity and representativeness of the sample may be insufficient. Future research should strive to expand the sample scope to enhance universality. Second, variable control may not be sufficient. The labeled social interaction mode of MBTI is regulated by many factors. In the future, more factors that may affect social anxiety should be introduced for deeper research. Looking ahead, a combination of multiple methods, cross-cultural comparative studies, and long-term tracking studies will help more comprehensively reveal the impact of using MBTI on social anxiety.

## Data Availability

The datasets presented in this study can be found in online repositories. The names of the repository/repositories and accession number(s) can be found in the article/Supplementary material.
