# Supplementary material for: From personality types to social labels: the impact of using MBTI on social anxiety among Chinese youth
Source: Front Psychol. 2024 Sep 4;15:1419492. doi: 10.3389/fpsyg.2024.1419492 (PMC11408848; doi:10.3389/fpsyg.2024.1419492)
Supplement: Supplementary file 1 [file Table_1.DOCX]

Supplementary Material

# Supplementary Data

Link: https://pan.baidu.com/s/1k0L-ccL3sUN1bzy_QhkMRQ?pwd=7l7w

Code: 7l7w

# Supplementary Figures and Tables

**Table 1**: Basic Information of Participants

| Basic Information | Category | Number | Percentage (%) | Cumulative Percentage  (%) |
| --- | --- | --- | --- | --- |
| Gender | Male | 247 | 52.7 | 52.7 |
|  | Female | 222 | 47.3 | 100.0 |
| Age Group | 18-25 years old | 137 | 29.2 | 29.2 |
|  | 26-30 years old | 205 | 43.7 | 72.9 |
|  | 31-35 years old | 127 | 27.1 | 100.0 |
| Education Level | Junior high school or below | 19 | 4.1 | 4.1 |
|  | High school/vocational school | 54 | 11.5 | 15.6 |
|  | Junior College | 147 | 31.3 | 46.9 |
|  | Undergraduate | 165 | 35.2 | 82.1 |
|  | Postgraduate or above | 84 | 17.9 | 100.0 |

**Table 2**: Reliability and Validity of Each Dimension of the Scale

| Variable | Cronbach’s Alpha | KMO | Bartlett’s Test of Sphericity |
| --- | --- | --- | --- |
| Using MBTI (UM) | 0.867 | 0.888 | sig.=0.000 |
| Ego Identity (EI) | 0.907 | 0.922 | sig.=0.000 |
| Belonging (B) | 0.892 | 0.935 | sig.=0.000 |
| Impression management (IM) | 0.913 | 0.977 | sig.=0.000 |
| Social anxiety (SA) | 0.955 | 0.974 | sig.=0.000 |

**Table 3**: Correlation Analysis Between Variables

| Variables | 1 | 2 | 3 | 4 | 5 |
| --- | --- | --- | --- | --- | --- |
| 1.Using MBTI（UM） | 1 |  |  |  |  |
| 2.Ego Identity（EI） | 0.754** | 1 |  |  |  |
| 3.Belonging（B） | 0.533** | 0.580** | 1 |  |  |
| 4.Impression management（IM） | 0.295** | 0.367** | 0.337** | 1 |  |
| 5.Social anxiety（SA） | 0.324** | 0.402** | 0.298** | 0.593** | 1 |

**Table 4**: Regression Analysis of Variable Relationships

| Regression Equation | | Overall Fit Index | | Significance of Regression Coefficients | |
| --- | --- | --- | --- | --- | --- |
| Dependent Variable | Independent Variable | R^2^ | F | β | t |
| EI | UM | 0.568 | 614.571 | 0.705 | 24.790*** |
| B | UM | 0.283 | 184.831 | 0.530 | 13.5953*** |
| IM | UM | 0.087 | 44.583 | 0.332 | 6.677*** |
| SA | UM | 0.390 | 74.415 | 0.035 | 0.474 |
|  | EI |  |  | 0.272 | 3.283** |
|  | B |  |  | -0.002 | -0.048 |
|  | IM |  |  | 0.600 | 13.029*** |

**Table 5**: Analysis of Mediation Effects

|  | Indirect effect value | Bootstrap SE | Bootstrapping 95% CI | | Relative indirect effects (%) |
| --- | --- | --- | --- | --- | --- |
|  |  |  | Lower-bound | Upper-bound |  |
| Total mediating effect | 0.390 | 0.072 | 0.249 | 0.530 | 91.80% |
| EI | 0.192 | 0.065 | 0.065 | 0.316 | 45.23% |
| B | -0.001 | 0.032 | -0.064 | 0.063 | -0.35% |
| IM | 0.199 | 0.029 | 0.142 | 0.258 | 46.92% |

## Supplementary Figures


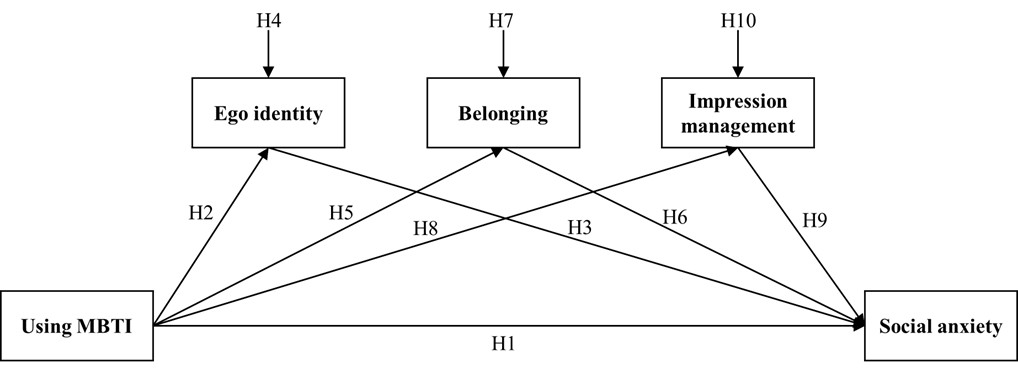


**Figure 1**: Hypotheses Proposed in the Study


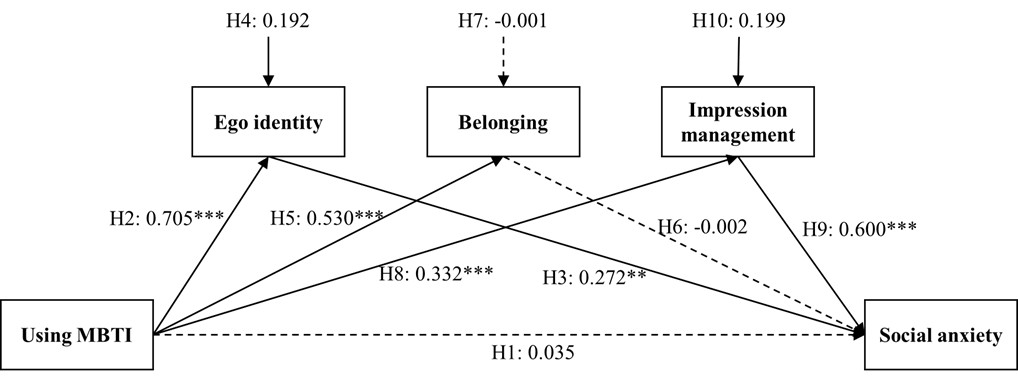


**Figure 2**: Model Diagram of the Association Between Using MBTI and Social Anxiety. Solid lines indicate supported hypotheses, and dashed lines indicate unsupported hypotheses. **p<0.01 ***p<0.001
